# Exploring the Impact of COVID-19 on Physical Activity One Month after Infection and Its Potential Determinants: Re-Infections, Pre-Illness Vaccination Profiles/Types, and Beyond

**DOI:** 10.3390/vaccines11091431

**Published:** 2023-08-29

**Authors:** Dimitrios I. Bourdas, Panteleimon Bakirtzoglou, Antonios K. Travlos, Vasileios Andrianopoulos, Emmanouil Zacharakis

**Affiliations:** 1 Section of Sport Medicine & Biology of Exercise, School of Physical Education and Sports Science, National and Kapodistrian University of Athens, 41 Ethnikis Antistasis, 17237 Daphne, Greece; dbourdas@phed.uoa.gr (D.I.B.); andrianopoulos.v@outlook.com (V.A.); emzach@phed.uoa.gr (E.Z.); 2 School of Physical Education and Sport Science, Aristotle University of Thessaloniki, 54124 Thessaloniki, Greece; 3 Department of Sports Organization and Management, Faculty of Human Movement and Quality of Life Sciences, University of Peloponnese, Efstathiou and Stamatikis Valioti & Plataion Avenue, 23100 Sparta, Greece; atravlos@uop.gr

**Keywords:** cross sectional, exercise, public health, Long COVID, mRNA, post-COVID-19 conditions (PCCs), post-acute Sequelae of SARS-CoV-2 infection (PASC), performance, retrospective, viral vector

## Abstract

This study investigated changes in physical activity (PA) after severe acute respiratory syndrome coronavirus 2 (SARS-CoV-2) infection while considering age, PA level, underlying medical conditions (UMCs), vaccination profiles/types, re-infections, disease severity, and treatment. Data were collected from 5829 respondents by using a validated web-based questionnaire. The findings showed that there was a significant overall decrease in PA (−16.2%), including in daily occupation (−11.9%), transportation (−13.5%), leisure-time (−16.4%), and sporting (−27.6%) activities. Age, PA level, UMCs, vaccination profiles/types, disease severity, and treatment played a role in determining PA in individuals’ post-acute SARS-CoV-2 infections. Re-infections did not impact the decline in PA. Unvaccinated individuals experienced a significant decline in PA (−13.7%). Younger (−22.4%) and older adults (−22.5%), those with higher PA levels (−20.6%), those with 2–5 UMCs (−23.1%), those who were vaccinated (−16.9%) or partially vaccinated (−19.1%), those with mRNA-type vaccines only (−17.1%), those with recurrent (−19.4%)-to-persistent (−54.2%) symptoms, and those that required hospital (−51.8%) or intensive care unit (−67.0%) admission during their infections had more pronounced declines in PA. These findings emphasize the complex relationship between post-acute SARS-CoV-2 infection and PA and highlight the need for targeted interventions, further research, and multidisciplinary care to promote PA resumption and mitigate long-term effects on global public health.

## 1. Introduction

The global establishment of COVID-19 [1], caused by the severe acute respiratory syndrome coronavirus 2 (SARS-CoV-2), has had a global impact and has affected various aspects of daily life including physical activity (PA) and sports. Emerging evidence suggests that SARS-CoV-2 infection is associated with an increased risk of acute and post-acute mortality [2,3,4,5], as well as with long-term consequences, which are known as Long COVID, and can persist for a period lasting from a few weeks (short-term) to more than six months (long-term) post-infection [6,7]. ‘Post-COVID-19 conditions’ (PCCs), a technical and interim term for Long COVID, encompass both the direct and indirect effects of SARS-CoV-2 infection on human health, and they lead to a wide range of sequelae, such as fatigue and respiratory, cardiovascular, neurological, and gastrointestinal symptoms that worsen with physical or mental exertion [6,8]. While the recovery from SARS-CoV-2 infection varies among individuals in terms of time and symptoms, re-infection further increases the risk of PCCs, which may lead to hospitalization and mortality in both the acute and post-acute phases [4,6]. Conversely, vaccination appears to increase the chances of prompt and complete recovery by reducing the risk of acute and post-acute sequelae, hospitalization, and mortality [2,9,10].

Fatigue, a common post-acute sequel of SARS-CoV-2 infection [11], either alone or in combination with other direct effects, may indirectly affect the PA levels of patients after infection. Recent research has demonstrated a significant impact of SARS-CoV-2 infection on exercise levels, particularly in terms of overall PA, among individuals in recreational sport activities [12]. The reductions in PA and daily energy expenditure associated with the post-acute sequelae of SARS-CoV-2 infection could contribute to the development of non-communicable diseases (NCDs) [13,14,15] and exacerbate sedentary behavior and negative psychological effects such as depression and anxiety [16]. This cascade effect has the potential to further burden patients who are recovering from SARS-CoV-2 infection and increase the risk of preventable long-term mortality. On the contrary, PA is related to improved mitochondrial metabolism in human cells through pAMPK and the reduction of pro-inflammatory cytokines via the activation of numerous signaling pathways through contraction-dependent signaling cascades [17]. Moreover, PA aids in the prevention and control of NCDs and hypertension, contributes to the upkeep of a favorable body weight, enhances psychological well-being, elevates the quality of life, and potentially serves as an indirect remedial measure against SARS-CoV-2 infection [18,19].

Individuals who have received full vaccination against SARS-CoV-2 and have subsequently become infected are less likely to experience symptoms of Long COVID compared to those who have received only one vaccine dose or remain unvaccinated [2,3,7,9,10]. However, the impact of pre-illness vaccination profile and type(s) on indirect post-acute sequelae, such as negative changes in PA, and the extent of protection against inactivity provided by prior vaccination remain unclear [3]. Additionally, the association between the incidence of SARS-CoV-2 re-infections and potential negative changes in PA after post-acute SARS-CoV-2 infection is not clearly understood. Furthermore, to the best of our knowledge, there is a lack of research on post-COVID sequelae and their impact on PA when specifically considering various domains such as daily occupation, transportation (to and from daily occupation), leisure-time, and sporting activities. The comprehensive assessment of changes in PA in the context of disrupted everyday life activities [20] necessitates the inclusion of all domains of PA. In addition, given the potential long-term implications for NCDs, healthcare systems, and public health, the reduction in PA during the COVID-19 era is a significant societal concern that necessitates further investigation. Thus, it is imperative to conduct additional research to address inactivity [21,22], examine risk factors and outcome measures for PCCs in diverse populations and settings [6,23], and develop comprehensive strategies for treating SARS-CoV-2 re-infections in both the acute and post-acute phases [24].

This study aims to assess the impact of post-acute SARS-CoV-2 infection on PA—both overall and in specific domains—one month after infection. We mainly examined the influence of pre-illness vaccination profile and type(s), incidence of re-infections, and other factors by using a validated web-based questionnaire. Our hypothesis is that the negative impact of post-acute SARS-CoV-2 infection on PA—both overall and in specific domains—is greater in the case of repeated infections and/or among unvaccinated individuals compared to that in those vaccinated against SARS-CoV-2. The current study primarily focused on assessing the relationships of post-acute SARS-CoV-2 infection, incidence of re-infections, and pre-illness vaccination profile and type(s) with PA levels rather than investigating the underlying biological mechanisms. The clinical implication of our study conveys valuable insights into the spectrum of Long COVID, particularly in relation to PA. The findings may provide important information for facilitating the optimal return to an active lifestyle for individuals who were infected with SARS-CoV-2. Additionally, this study could generate scientific data that can inform the development of effective strategies for supporting physical activity and preventing inactivity, thereby reducing the risk of NCDs [25,26,27,28].

## 2. Materials and Methods

**Figure 1:**
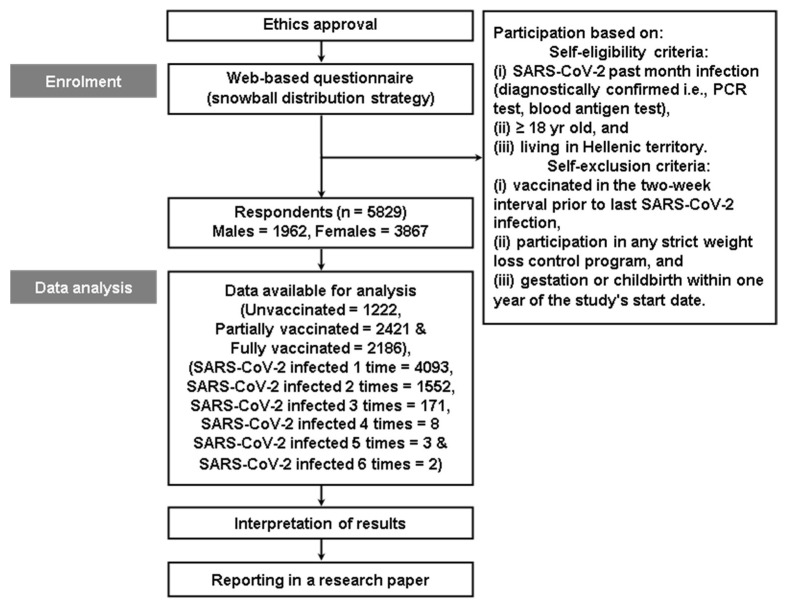
Study design. Abbreviation: SARS-CoV-2, severe acute respiratory syndrome coronavirus 2.

Links to a comprehensive multi-dimensional web-based questionnaire were electronically distributed for the needs of this study. The self-eligibility criteria included a confirmed SARS-CoV-2 infection within the last 30–40 days (confirmed by diagnostic tests such as polymerase chain reaction or blood antigen tests); the participants needed to be at least 18 years old, and they needed to be residents of the Hellenic territory (Greece). Self-exclusion criteria consisted of recent vaccination within the two weeks prior to the last SARS-CoV-2 infection, participation in strict weight loss programs, and recent gestation or childbirth within one year of the study’s start date. The study (Figure 1) was approved by the local University Ethics Committee (protocol number: 1454/11-01-2023, ClinicalTrials.gov identifier: NCT05787431). Written information about the study was provided to potential participants, and informed consent was obtained from those who voluntarily agreed to participate.

### 2.1. Survey Instrument

Active-Q [29,30], a self-reported online questionnaire, was used to assess the weekly habitual physical activity of adults in various domains (daily occupation, transportation (to and from daily occupation), leisure-time, and sporting activities). In addition to Active-Q, simple questions (i.e., items) were also incorporated. Five questions were on participants’ physical characteristics (ethnic origin, sex at birth, age, height, and weight), one was about their daily smoking habits, one was about their region of residence (urban proximity), and one was on their education level [31]. Regarding the existence of certain underlying medical conditions (UMCs) in the participants that could be related to severe illness with COVID-19 (e.g., cancer, chronic kidney disease, cystic fibrosis, tuberculosis, diabetes, neurocognitive disorders, essential hypertension, chronic heart disease, chronic liver disease, chronic lung disease, stroke or cerebrovascular disease, organ transplant recipient, substance use disorders, sickle cell anemia or thalassemia, HIV, ≥65 years of age, obesity, physical disabilities, smoking), one question (yes or no) and a follow-up question on the UMCs (yes or no) were included in order to compute the number of specific conditions. With reference to the pre-illness vaccination profiles, one to nine questions (depending on the previous responses and follow-up questions) on each participant’s pre-illness vaccination status (vaccinated/unvaccinated, vaccine type(s), number of administered doses, time to infection from the last vaccine dose, and/or time between last two vaccine doses if they were applicable) were asked. To assess the incidence of SARS-CoV-2 re-infections, participants were asked about their frequency of occurrence. Coronavirus disease severity was evaluated by asking participants about the frequency [12] of their primary complaints (some of them) following SARS-CoV-2 infection (e.g., fever: >38 °C, dyspnea, chills, cough, sore throat, generalized body aches, chest pain, abdominal pain, back pain, joint pain, headache, weakness, fatigue, altered mental status, diarrhea, vomiting, loss of smell and/or taste, chills) [32]. Regarding (illness) treatment during SARS-CoV-2 infection, one question on certain treatments was asked (yes or no).

All questions, except for the questionnaire items regarding anthropometric characteristics and the incidence of re-infections, had a fixed set of answers [30,33]. All of the questions incorporated in addition to Active-Q were created in an ad hoc manner and were not validated on their own; for that reason, a pilot study (*n* = 31) was conducted to ensure that the questions were comprehensible for the sample population, as suggested in a recent study [12]. The original version of Active-Q was validated against doubly labeled water and an accelerometer [29,34], whereas the current submission form version was previously tested for reliability [30], and this is presented in full detail elsewhere [33]. In total, the web-based questionnaire consisted of 60–67 questions based on the previous responses and follow-up patterns. Moreover, by utilizing an added item (reCAPTCHA v3), each potential participant had to prove that they were not a robot prior to submitting the answered questionnaire. In addition, to avoid multiple enrolments from the same individual as much as possible, restriction through personal email addresses (that were not recorded in the response sheet or by any other means) was applied, resulting in there not being multiple responses from the same email addresses.

### 2.2. Data Acquisition Procedure

Potential study participants were openly invited by using a snowball distribution strategy that included social media links, emails, and/or nationwide public advertisements. The web-based questionnaire required participants to complete Active-Q twice, providing information on their PA levels one to two weeks before (PRE condition) and one month after their last SARS-CoV-2 infection (POST condition), in addition to the aforementioned questions. The time frame of the study (February to the end of March 2023, i.e., two months) was predetermined, and beyond this time period, we did not accept any further submissions. The PAs were categorized according to the Compendium of Physical Activities, with each being assigned a corresponding metabolic equivalent task (MET = 3.5 mL O_2_·kg^−1^·min^−1^) value [30,35]. The total time spent on PA in each domain was reported, and the energy expenditure in METs was automatically calculated (min·week^−1^). The participants’ disease severity was automatically determined by calculating the frequency of reported symptoms and dividing it by the total number of complaints [12].

Based on their responses, the 5829 volunteer respondents (ethnic origin: 5789 (99.3%) Caucasian and 40 (0.7%) other; age: 45.64 ± 10.35 yr [45.4–45.9]; weight: 75.7 ± 17.1 kg [75.3–76.2]; height: 170.0 ± 9.0 cm [169.7–170.2]; body mass index (BMI): 26.1 ± 5.0 kg·m^−2^ [26.0–26.2]) were divided into two groups for sex at birth, five for age, four for BMI, four for PA level, four for smoking status, three for region of residence, seven for education level, five for UMCs [36], six for incidence of SARS-CoV-2 re-infections, four for pre-illness vaccination profile, five for pre-illness vaccine type(s) received, five for disease severity, and five for illness treatment subgroup (see the Section 3). Participants’ PA levels and BMI subgroups were determined based on their pre-SARS-CoV-2 infection statuses. The vaccine doses administered in the sample population included various combinations. For the purposes of this study, participants who had received two or more doses of messenger ribonucleic acid (mRNA)-type or protein subunit (PS)-type vaccines, one dose of a viral vector (VV)-type vaccine, or a heterologous vaccine combination at least six months prior to their last SARS-CoV-2 infection were classified as “fully vaccinated”. Participants who had received only one dose of a multi-dose COVID-19 vaccine series or had completed the primary course but had had time intervals of longer than six months between their last doses or boosters and their last SARS-CoV-2 infections were classified as “partially vaccinated”. Both partially and fully vaccinated participants were considered “vaccinated” (i.e., individuals who had received at least one vaccine dose).

### 2.3. Data Analysis

Unless otherwise specified, the frequency and/or relative frequency (%) of each of the qualitative variables and the mean, standard deviation (SD), and 95% confidence interval (CI) of each of the quantitative variables are reported. To compare quantitative variables (i.e., overall PA, domain-specific activities) within the same subgroup (i.e., by respondent sex at birth, age, BMI, PA level, smoking status, residence region, education level, UMCs, vaccination profile and type(s), incidence of SARS-CoV-2 re-infections, disease severity, and illness treatment) under the two conditions (i.e., PRE and POST), paired t-tests were performed [37]. One-way ANCOVA was employed to examine whether there were any significant differences in the POST condition’s PA-adjusted mean (i.e., the PRE illness condition was used as a covariate) between the different subgroups. In cases where a significant difference was discovered, post hoc analysis (Bonferroni pairwise comparisons) was performed [37]. The Statistical Package for the Social Sciences for the Windows platform (SPSS 29.0, IBM Corp, Armonk, NY, USA) was used to analyze all of the data in accordance with Meyers and colleagues’ procedures [38]. The statistical significance level was predetermined at *p* ≤ 0.05 for all analyses. Moreover, since no previous research with a similar experimental design had been undertaken, a post hoc power analysis was performed using the G*Power software (version 3.1.9.2, Heinrich-Heine-University Dusseldorf, Dusseldorf, Germany) with the overall PA as a criterion variable. For the power analysis, the following parameters were used: α = 0.05, an effect size of 0.20, a sample size of 5829, and one group in two conditions. The observed power (1-β) was greater than 0.99. A similar power was calculated when the power variables, such as daily energy expenditure for occupational, transportation, and sporting activities, were used.

## 3. Results

Of the total respondents, 1962 (33.7%) were males (age: 47.5 ± 10.8 yr [47.0–48.0], weight: 88.3 ± 15.0 kg [87.6–88.9], height: 178.7 ± 7.3 cm [178.3–179.0], BMI: 27.6 ± 4.4 kg·m^−2^ [27.4–27.8]) and 3867 (66.3%) were females (age: 44.7 ± 10.0 yr [44.4–45.0], weight: 69.4 ± 14.3 kg [68.9–69.8], height: 165.5 ± 6.1 cm [165.3–165.7], BMI: 25.3 ± 5.1 kg·m^−2^ [25.1–25.5]). The outcomes for PA are displayed for the different subgroups under both the PRE and POST conditions; Table 1 focuses on the overall PA estimations, while the domain-specific activities are provided in Table 2. As illustrated in Table 1, under the POST condition, there was a significant decrease in overall PA within the study population and in the vast majority of the subgroups (*p* ≤ 0.05 for all) in comparison with that under the PRE condition. Similarly, under the POST condition, the energy expenditure associated with daily occupational, transportation, and sporting activities significantly decreased within the study population and in the vast majority of subgroups (*p* ≤ 0.05 for all) in comparison with that under the PRE condition (Table 2).

Moreover, the application of one-way ANCOVA tests and post hoc analysis indicated that there was statistical significance (*p* ≤ 0.05) between various subgroups in terms of the overall PA and domain-specific activities (Table 3 and Table 4). Briefly, the significant impacts of factors such as age, PA level, number of UMCs, pre-illness vaccination profile and type(s), disease severity, and illness treatment on overall PA and domain-specific activities were identified (Table 3 and Table 4). Statistical significance (*p* ≤ 0.05) was also observed for respondents who were younger, had higher levels of PA, had 2–5 UMCs, were vaccinated, had received partial vaccination, had received mRNA-type vaccines only, had experienced recurrent-to-persistent symptoms during their SARS-CoV-2 infections, or required hospital or ICU admission (Table 3 and Table 4). Additionally, there was a trend (*p* = 0.09) indicating the potential significance of the relationships of the “unvaccinated” and “fully vaccinated” subgroups with overall PA (Table 3). On the contrary, respondent sex at birth, BMI, smoking status, education level, pre-illness vaccine type(s) (in the vaccinated population), and incidence of SARS-CoV-2 re-infections were not significant (*p* > 0.05) with respect to the overall PA (Table 3); nor were the respondents’ vaccine type(s) significant with respect to the leisure-time activities or incidence of SARS-CoV-2 re-infections with respect to the activity domains (Table 4).

**Figure 2:**
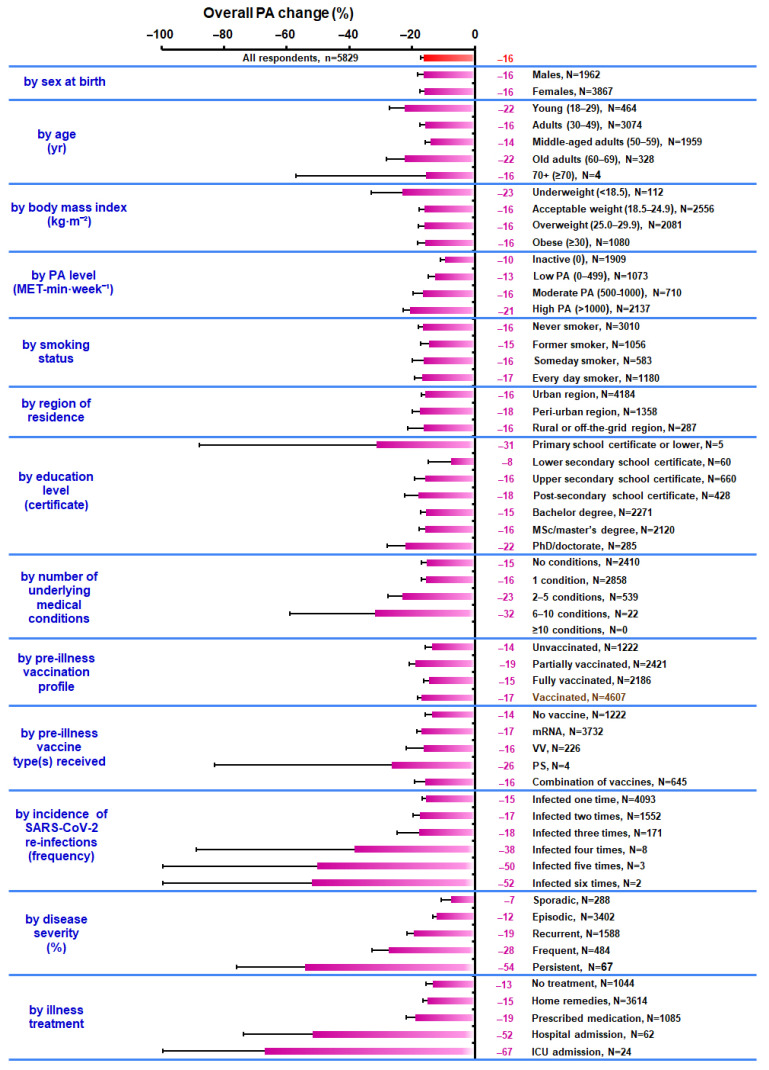
Change in overall PA (%, from the PRE to the POST conditions) on a weekly basis in all participants and in the subgroups according to respondent sex at birth, age, body mass index, PA level, smoking status, region of residence, education level, number of underlying medical conditions, pre-illness vaccination profile, pre-illness vaccine type(s) received, incidence of SARS-CoV-2 re-infections, disease severity, and illness treatment. Error bars present the lower bounds of the 95% confidence intervals. Abbreviations: ICU, intensive care unit; MET, metabolic equivalent task (1 MET = 3.5 mL O_2_·kg^−1^·min^−1^); mRNA, messenger ribonucleic acid; n, sample size; N, subgroup’s sample size; PA, physical activity; POST, one month post-SARS-CoV-2 infection; PRE, 1–2 wk pre-SARS-CoV-2 infection; PS, protein subunit; SARS-CoV-2, severe acute respiratory syndrome coronavirus 2; VV, viral vector.

**Figure 3:**
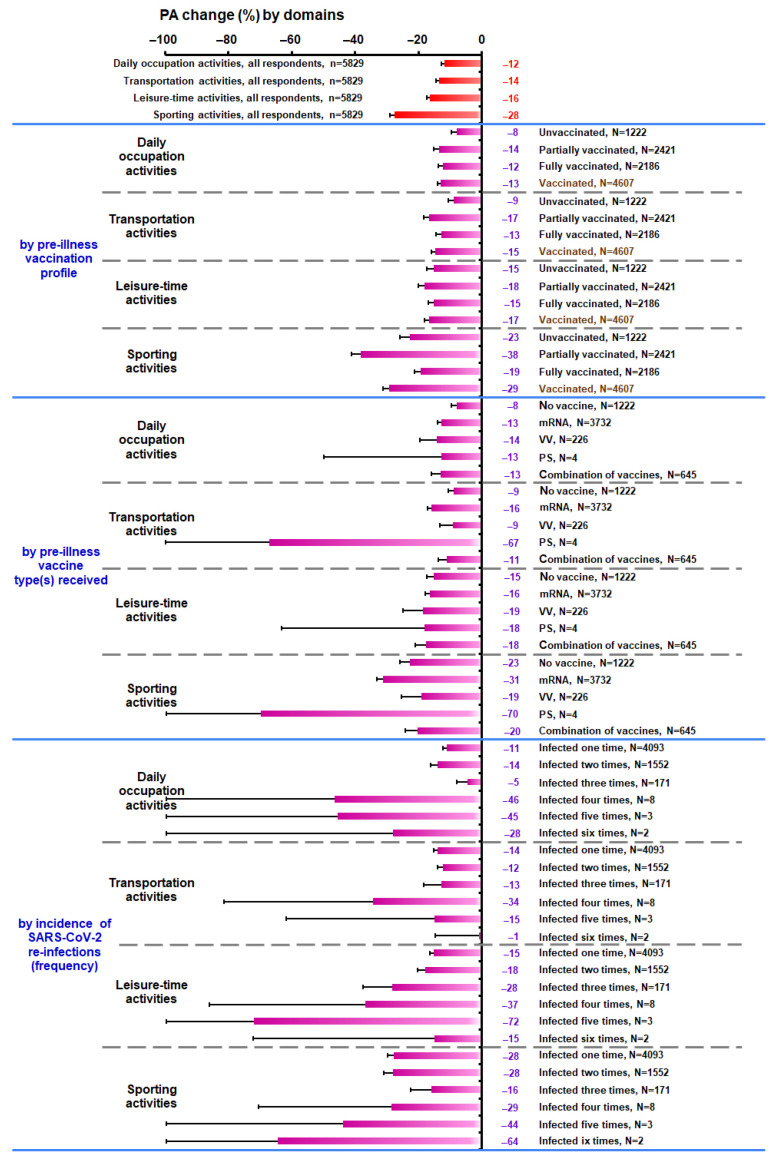
Change in daily occupation, transportation (to and from daily occupation), leisure-time, and regular sporting activities (%, from the PRE to POST conditions) on a weekly basis in all participants and in the subgroups according to respondent pre-illness vaccination profile, pre-illness vaccine type(s) received, and incidence of SARS-CoV-2 re-infections. Error bars present the lower bounds of the 95% confidence intervals. Abbreviations: MET, metabolic equivalent task (1 MET = 3.5 mL O_2_·kg^−1^·min^−1^); mRNA, messenger ribonucleic acid; n, sample size; N, subgroup’s sample size; PA, physical activity; POST, one month post-SARS-CoV-2 infection; PRE, 1–2 wk pre-SARS-CoV-2 infection; PS, protein subunit; SARS-CoV-2, severe acute respiratory syndrome coronavirus 2; VV, viral vector.

Figure 2 and Figure 3 depict the weekly changes (%) in overall PA and in domain-specific activities, respectively from the PRE to the POST conditions for all respondents and the various subgroups. Notably, the 95% CI for the change in overall PA ranged from −17.3% to −15.1% among all respondents, from −15.9% to −11.5% among the unvaccinated, from −21.0% to −17.2% among the partially vaccinated, from −16.2% to −12.8% among the fully vaccinated, from −18.2% to −15.6% among the vaccinated, and from −18.5% to −15.7% among respondents who had been vaccinated with mRNA-type vaccines only, whereas it ranged from −27.2% to −17.7% among “young” respondents, from −22.7% to −18.5% among respondents with “high PA”, from −27.6% to −18.6% among respondents with “2–5 conditions”, from −73.8% to −29.7% among respondents with “hospital admission”, from −100.0% to −24.7% among respondents with “ICU admission”, from −21.7% to −17.0% among respondents in the “recurrent” subgroup, and from −76.0% to −32.3% among respondents in the “persistent” subgroup (Table 3, Figure 2). Furthermore, the changes in PA revealed several distinct patterns in daily occupational, transportation, and sporting activities in the “unvaccinated” and “fully vaccinated” or “partially vaccinated” subgroups and among unvaccinated respondents and respondents who had been vaccinated with mRNA-type vaccines only (Table 4, Figure 3).

## 4. Discussion

This study examined the impact of post-acute SARS-CoV-2 infection on PA in relation to re-infection incidence, pre-illness vaccination profile and type(s), and other factors. We reported a significant decrease in overall PA per week (−16.2%), which was also reflected in domain-specific activities including daily occupation (−11.9%), transportation (−13.5%), leisure-time (−16.4%), and sports (−27.6%). Age, PA level, number of underlying medical conditions, pre-illness vaccination profile and type(s), disease severity, and illness treatment were strongly associated with changes in PA. However, the incidence of re-infections did not have a significant impact on the decline in overall PA. Younger and older adults, those with higher PA levels, individuals with 2–5 underlying medical conditions, vaccinated individuals, those who had received partial vaccination or mRNA-type vaccines only, individuals with recurrent-to-persistent symptoms, and those who required hospital or ICU admission during their infection experienced the most substantial decline in overall PA.

In the Hellenic territory, as of the end of February 2023, 73.1% of residents had received at least one dose of a COVID-19 vaccine [39]. During the period to which this study’s data correspond, 7.3% of the confirmed positive SARS-CoV-2 cases sought hospital assistance or were hospitalized, and 0.32% were admitted to ICUs [39]. These percentages align with our study findings, suggesting the representativeness of our study population in these aspects.

Previous evidence suggested a decline in exercise levels among recreational athletes (*n* = 601) by 58.0% one month after SARS-CoV-2 infection [12] and a delayed return to sports after positive SARS-CoV-2 infection for elite athletes (*n* = 147) [40]. In our study, a significant decrease in overall PA and domain-specific activities was observed in most subgroups one month after SARS-CoV-2 infection, independently of anthropometric traits, PA level, smoking status, region of residence, education level, number of underlying medical conditions, pre-illness vaccination profile and type(s), incidence of SARS-CoV-2 re-infections, disease severity, and illness treatment. The decline in PA following post-acute SARS-CoV-2 infection can be attributed to factors such as autonomic dysfunction [41] and/or guidelines recommending a period of asymptomatic recovery before resuming exercise, as well as to the anxiety associated with restarting physical activity after an infection [12]. Additionally, the symptoms and recovery process associated with the infection, including fatigue, weakness, and respiratory difficulties, may contribute to decreased participation in PAs [2]. Our findings also suggest that increased disease severity and advanced medical intervention are associated with a more pronounced decline in overall PA and domain-specific activities [4]. This relationship can be explained by the physical limitations experienced by individuals with more severe illnesses and the extended periods of rest and recovery required during intensive medical interventions.

Several factors, including a higher BMI, female sex at birth, smoking, and older age, have been identified as being associated with an increased risk of developing symptoms of Long COVID [42,43]. However, in our sample population, factors such as sex at birth, BMI, smoking status, region of residence, and education level did not show significant associations with the decline in PA following acute SARS-CoV-2 infection. Conversely, compared to adults or middle-aged adults, young individuals, as well as highly physically active individuals in comparison with moderately active individuals, experienced significant reductions in overall PA, which were possibly due to a decrease in leisure-time and/or sports activities following post-acute SARS-CoV-2 infection.

Likely, previous exposure to SARS-CoV-2 reduces the risk of re-infection and its severity due to increased natural immunity [44,45]. However, in the current study, 29.8% of the participants had experienced recurring SARS-CoV-2 infections, which were associated with decreases in overall PA and domain-specific activities. In addition, the incidence of re-infections did not show a significant association with the decline in PA following acute SARS-CoV-2 infection. Another study demonstrated that re-infection with SARS-CoV-2 increased the risk of death and negative health consequences during both the initial and subsequent phases of re-infection [4]. Moreover, SARS-CoV-2 undergoes frequent mutations, with novel variants such as Omicron exhibiting a high capacity for evading immunity from previous infections, resulting in an elevated risk of re-infection [46,47]. Additionally, the protective effects of previous infections diminish over time and are independent from vaccination status [46,48]. Individuals who experience health impairment following an initial SARS-CoV-2 infection may be at a higher risk of developing health issues and sequelae upon subsequent re-infection [46,48]. Consequently, the potential severity of re-infections may not facilitate conditions for mitigating the impact of viral infection on the reduction in PA.

COVID-19 vaccines have been shown to effectively prevent SARS-CoV-2 infections by inducing antibody production and conferring immunity [49]. However, they can also cause adverse local and systemic effects such as fatigue, headache, fever, pain, and tenderness at the injection site [50], with lower incidence rates being reported for mRNA-type vaccines [51]. In our study, individuals who were highly physically active, those who were vaccinated or partially vaccinated, and those who had received mRNA-type vaccines only (which were predominant among vaccine recipients) showed a higher likelihood of experiencing reduced PA one month after acute SARS-CoV-2 infection. Moreover, a separate study on young students (18–23 yr) without prior SARS-CoV-2 infection found that the physically active group (*n* = 20) exhibited a significant decrease in antibody levels (IgG and IgM) after three doses of mRNA-type vaccines, while the less active group (*n* = 20) showed increased antibody levels after one month [52]. This phenomenon could be attributed to the interaction between mRNA-type vaccines and the musculoskeletal system, potentially resulting in vaccine degradation after heightened PAs. It is worth noting that higher levels of PA may be associated with a decline in antibody levels following the administration of an mRNA-type vaccine [53]. Several factors contribute to this association, including compromised immune systems in individuals with higher susceptibility to infections, such as respiratory tract infections, which may limit their ability to engage in PAs. Additionally, a weakened immune system can lead to fatigue, muscle weakness, and a decreased in overall physical capacity [28,54,55]. Our findings seem to align with evidence suggesting a potential compromise in the working mechanism of mRNA-type vaccines among highly physically active individuals [53]. Furthermore, the combined effect of vaccination and SARS-CoV-2 infection itself may create a potent combination that further compromises PA levels. On the other hand, the mechanisms involved in the immune response to vaccines and their effects on PA may vary depending on the pre-illness vaccine type(s) and individual factors. Full COVID-19 vaccination has been shown not only to prevent symptomatic infection, but also to potentially mitigate the risk of Long COVID [53]. Evidence suggests that in certain individuals with Long COVID, symptoms may be ameliorated following the administration of one or both doses of a COVID-19 vaccination [56]. Furthermore, it has been observed that regular physical exercise in adults can enhance the antibody response to influenza or COVID-19 vaccination when followed by a single session of light- to moderate-intensity exercise after immunization [57]. However, the association found between the perceived pressure to undergo full vaccination and the negative alteration in physical performance perceived among elite athletes (*n* = 306) indicates the psychological influence of perceived pressure as a determinant [58]. Therefore, these divergent findings necessitate additional research on the relationship between vaccines and PA.

### Limitations, Strengths, and Recommendations for Future Research

The generalizability of our study’s findings is limited to the adult population residing in the Hellenic territory, and the vaccine combinations were decided based on the recommendations of doctors, personal preferences, and the vaccines’ availability [59]. Thus, it was not possible to control for additional vaccine doses that were administered as an extension of the primary course in the sample population (e.g., in immunocompromised respondents), heterologous vaccine schemes (e.g., when vaccination with a second dose with the same vaccine was medically contraindicated), and extra boosters (e.g., in vulnerable individuals). Given the coexistence of numerous variants of SARS-CoV-2, controlling for specific variants was not feasible. It was also possible that some respondents had asymptomatic infections that were not tested, leading to the potential misclassification of SARS-CoV-2 exposure. Another limitation was the limited timeframe for assessing the impact of post-acute SARS-CoV-2 infection on PA, as the long-term health consequences may not have been captured [2]. Our findings may be limited to individuals who did not have prolonged ICU hospitalization or fatal outcomes. This study was conducted online, which may have introduced bias towards those with internet access [60], thus potentially overrepresenting certain demographic groups. The measurement of PA relied on self-reporting, which may have been subject to recall bias. The sample sizes in certain subgroups were small, thus limiting the generalizability of the findings. The differences observed in the impact of post-acute SARS-CoV-2 infection on different domain-specific activities—particularly sporting activities—may have been influenced by psychological, socioeconomic, nutritional, or daily priorities, but these factors were not controlled for in our study. Herein, we recognize the protective role of some nutrients, such as quercetin, in COVID-19 progression; quercetin exerts antiviral activity and is able to reduce interleukin 1 and 6 in human plasma with several beneficial effects [61]. Moreover, arginine-related pathways in COVID-19 diseases, which are based on the T cell function dependence on l-arginine levels, may have determined, to some extent, the levels of PA [62], although they were not controlled by the current study.

Despite the limitations, the current study makes a valuable contribution to the field as it is the first of its kind to comprehensively examine the impact of post-acute SARS-CoV-2 infection on changes in PA by taking various factors, such as pre-illness vaccination profile and type(s), incidence of re-infections, and other relevant parameters, into account. This investigation provides deep insight into the assessment of PA in adults that have recovered from SARS-CoV-2, as it has included the evaluation of four important activity domains and generated hypotheses for future research in the context of disruptions in the activities of everyday life.

While COVID-19 vaccines offer promise in combatting SARS-CoV-2 infection and restoring normalcy, there is a lack of research on the influences of re-infections, pre-illness vaccination profile and type(s), and other factors on PA following post-acute SARS-CoV-2 infection. The effectiveness of COVID-19 vaccines with respect to the securing PA in adults that have recovered from SARS-CoV-2 remains unclear, highlighting the need for further investigation. Additionally, comprehensive clinical trials assessing specialized training protocols for the resumption of PA in individuals with post-acute SARS-CoV-2 infection are crucial, and they must consider factors such as vaccination profile, immune system status, and diverse populations. These efforts enhance our understanding of effective interventions for managing Long COVID. Undoubtedly, all of these areas necessitate additional research efforts and funding support.

## 5. Conclusions

In summary, our study revealed a significant decline in overall PA and domain-specific activities following post-acute SARS-CoV-2 infection. Factors such as age, PA level, number of underlying medical conditions, pre-illness vaccination profile and type(s), disease severity, and illness treatment influenced the level of PA following post-acute SARS-CoV-2 infection. Unvaccinated individuals experienced notable reductions in PA, but the most pronounced declines were observed in younger and older adults, those with higher PA levels, individuals with 2–5 underlying medical conditions, vaccinated individuals, those who had received partial vaccination or exclusively mRNA-type vaccines, individuals with recurrent-to-persistent symptoms, and those requiring hospital or ICU admission during their SARS-CoV-2 infection. These findings highlight the complex relationships among pre-illness vaccination profile and type(s), individual characteristics, and PA levels, underscoring the need for targeted interventions and further research to promote PA among individuals recovering from post-acute SARS-CoV-2 infection. Nonetheless, the best approach to preventing and mitigating the sequelae of post-COVID-19 conditions is to avoid exposure to the virus through vaccination, physical distancing, respiratory protection, and adherence to basic hygiene practices.

## Figures and Tables

**Table 1:** Overall PA statistics presented as means ± SD [95% CI] according to respondent sex at birth, age, body mass index, PA level, smoking status, region of residence, education level, number of underlying medical conditions, vaccination profile, vaccine type(s) received, incidence of SARS-CoV-2 re-infections, disease severity ^†^, and illness treatment under the PRE and POST conditions.

Variable	Subgroup, Frequency (%)	PRE, (MET-Min·Week^−1^)	POST, (MET-Min·Week^−1^)
----	All respondents * 5821 (100.0)	15,256.3 ± 12,719.3 [14,929.8–15,582.8]	12,788.6 ± 10,682.2 [12,514.3–13,062.8]
Sex at birth	Males *, 1962 (33.7)	15,775.8 ± 14,015.1 [15,155.7–16,396.0]	13,192.8 ± 11,296.8 [12,692.9–13,692.6]
	Females *, 3867 (66.3)	14,992.7 ± 12,001.7 [14,614.4–15,370.9]	12,583.5 ± 10,351.9 [12,257.2–12,909.8]
Age (yr)	Young (18–29) *, 464 (8.0)	15,253.1 ± 11,127.0 [14,240.6–16,265.5]	11,830.3 ± 8927.7 [11,018.0–12,642.6]
	Adults (30–49) *, 3074 (52.7)	15,400.0 ± 14,000.3 [14,905.0–15,894.8]	12,955.5 ± 11,213.1 [12,559.1–13,351.9]
	Middle-aged adults (50–59) * 1959 (33.6)	15,094.5 ± 10,867.8 [14,613.3–15,575.8]	12,964.2 ± 10,341.4 [12,506.3–13,422.2]
	Old adults (60–69) *, 328 (5.6)	14,965.4 ± 12,557.1 [13,606.4–16,324.3]	11,600.9 ± 9757.8 [10,544.9–12,657.0]
	70+ (≥70) *, 4 (0.1)	8283.5 ± 3399.1 [4952.5–11,614.5]	6992.0 ± 3728.3 [3338.3–10,645.7]
Body mass index (kg·m^−2^)	Underweight (<18.5) *, 112 (1.9)	18,075.3 ± 13,124.1 [15,644.7–20,505.8]	13,882.2 ± 9170.6 [12,183.8–15,580.6]
	Acceptable weight (18.5–24.9) *, 2556 (43.8)	15,668.6 ± 11,287.0 [15,231.0–16,106.1]	13,159.5 ± 9918.2 [12,775.0–13,544.0]
	Overweight (25.0–29.9) *, 2081 (35.7)	15,018.0 ± 12,051.0 [14,500.2–15,535.7]	12,597.2 ± 9598.3 [12,184.9–13,009.6]
	Obese (≥30) *, 1080 (18.5)	14,447.4 ± 16,507.1 [13,462.9–15,431.9]	12,166.0 ± 14,008.6 [11,330.5–13,001.4]
PA level (MET-min·week^−1^)	Inactive (0) *, 1909 (32.7)	11,957.7 ± 8056.7 [11,596.3–12,319.1]	10,820.2 ± 7347.7 [10,490.6–11,149.9]
	Low PA (0–499) *, 1073 (18.4)	12,117.9 ± 7321.9 [11,679.8–12,556.0]	10,580.6 ± 6807.8 [10,173.3–10,988.0]
	Moderate PA (500–1000) *, 710 (12.2)	12,998.1 ± 6331.8 [12,532.4–13,463.9]	10,862.6 ± 6097.2 [10,414.1–11,311.1]
	High PA (>1000) *, 2137 (36.7)	20,529.0 ± 17,292.6 [19,795.8–21,262.1]	16,295.4 ± 14,424.5 [15,683.8–16,907.0]
Smoking status	Never smoker *, 3010 (51.6)	15,458.8 ± 13,463.1 [14,977.9–15,939.8]	12,918.5 ± 12,059.1 [12,487.7–13,349.3]
	Former smoker *, 1056 (18.1)	14,542.0 ± 11,658.9 [13,838.8–15,245.2]	12,404.5 ± 8380.6 [11,899.1–12,910.0]
	Someday smoker *, 583 (10.0)	15,547.3 ± 11,078.9 [14,648.0–16,446.6]	13,007.6 ± 9286.7 [12,253.8–13,761.4]
	Every day smoker *, 1180 (20.2)	15,235.0 ± 12,421.0 [14,526.3–15,943.7]	12,692.6 ± 9342.9 [12,159.6–13,225.7]
Region of residence	Urban region *, 4184 (71.8)	15,473.4 ± 13,250.9 [15,071.9–15,874.9]	13,040.7 ± 11,412.2 [12,694.9–13,386.5]
	Peri-urban region *, 1358 (23.3)	14,612.5 ± 10,690.1 [14,044.0–15,181.1]	12,038.4 ± 8461.1 [11,588.4–12,488.4]
	Rural or off-the-grid region *, 287 (4.9)	15,137.3 ± 13,552.5 [13,569.3–16,705.2]	12,662.4 ± 8780.7 [11,646.5–13,678.2]
Education level (certificate)	Primary school certificate or lower, 5 (0.1)	40,226.9 ± 32,625.1 [11,630.2–68,823.5]	27,574.2 ± 16,971.1 [12,698.7–42,449.8]
	Lower secondary school certificate, 60 (1.0)	17,566.6 ± 13,708.1 [14,098.1–21,035.2]	16,239.8 ± 18,440.4 [11,573.8–20,905.8]
	Upper secondary school certificate *, 660 (11.3)	16,937.7 ± 12,559.2 [15,979.5–17,895.8]	14,241.5 ± 11,120.8 [13,393.1–15,089.9]
	Post-secondary school certificate *, 428 (7.3)	15,701.8 ± 10,757.5 [14,682.7–16,721.0]	12,886.4 ± 8805.6 [12,052.2–13,720.6]
	Bachelor degree *, 2271 (39.0)	14,719.2 ± 11,530.8 [14,245.0–15,193.4]	12,444.6 ± 9484.5 [12,054.5–12,834.6]
	MSc/master’s degree *, 2120 (36.4)	14,763.6 ± 11,442.8 [14,276.5–15,250.7]	12,415.2 ± 10,263.7 [11,978.3–12,852.1]
	PhD/doctorate *, 285 (4.89)	17,713.1 ± 25,632.1 [14,737.3–20,689.0]	13,809.4 ± 18,548.5 [11,656.0–15,962.9]
Number of underlying medical conditions	No conditions *, 2410 (41.3)	15,570.8 ± 11,211.3 [15,123.2–16,018.4]	13,190.4 ± 10,285.9 [12,779.8–13,601.1]
	1 condition *, 2858 (49.0)	15,126.1 ± 14,198.9 [14,605.6–15,646.7]	12,769.4 ± 11,338.2 [12,353.7–13,185.1]
	2–5 conditions *, 539 (9.2)	14,647.4 ± 10,775.1 [13,737.7–15,557.0]	11,264.1 ± 8609.1 [10,537.4–11,990.9]
	6–10 conditions *, 22 (0.4)	12,631.0 ± 6766.1 [9803.7–15,458.3]	8606.0 ± 6787.3 [5769.9–11,442.2]
	≥10 conditions, 0 (0.0)	----	----
Pre-illness vaccination profile	Unvaccinated *, 1222 (21.0)	16,655.2 ± 16,783.1 [15,714.2–17,596.2]	14,375.6 ± 14,754.9 [13,548.3–15,202.8]
	Partially vaccinated *, 2421 (41.5)	14,811.5 ± 12,130.0 [14,328.3–15,294.7]	11,985.0 ± 8460.0 [11,648.0–12,322.0]
	Fully vaccinated *, 2186 (37.5)	14,966.9 ± 10,476.1 [14,527.7–15,406.0]	12,791.4 ± 10,065.3 [12,369.4–13,213.3]
	Vaccinated *, 4607 (79.0)	14,885.2 ± 11,374.3 [14,556.8–15,213.7]	12,367.6 ± 9264.2 [12,100.1–12,635.1]
Pre-illness vaccine type(s) received	No vaccine *, 1222 (21.0)	16,655.2 ± 16,783.1 [15,714.2–17,596.2]	14,375.6 ± 14,754.9 [13,548.3–15,202.8]
	mRNA *, 3732 (64.0)	14,989.1 ± 11,800.9 [14,610.5–15,367.7]	12,424.6 ± 9471.0 [12,120.7–12,728.4]
	VV *, 226 (3.9)	14,549.3 ± 8687.4 [13,416.7–15,681.9]	12,179.0 ± 7595.3 [11,188.8–13,169.3]
	PS, 4 (0.1)	18,213.2 ± 2840.5 [15,429.6–20,996.9]	13,406.0 ± 2033.0 [11,413.7–15,398.3]
	Combination of vaccines *, 645 (11.1)	14,381.1 ± 9574.6 [13,642.2–15,120.1]	12,097.8 ± 8594.9 [11,434.5–12,761.1]
Incidence of SARS-CoV-2 re-infections (frequency)	Infected one time *, 4093 (70.2)	15,044.6 ± 13,489.3 [14,631.3–15,457.8]	12,719.4 ± 11,013.5 [12,382.0–13,056.9]
	Infected two times *, 1552 (26.6)	15,523.0 ± 10,458.3 [15,002.6–16,043.3]	12,809.2 ± 9515.9 [12,335.8–13,282.6]
	Infected three times *, 171 (2.9)	17,478.1 ± 12,276.8 [15,638.0–19,318.2]	14,359.7 ± 12,683.2 [12,458.7–16,260.7]
	Infected four times, 8 (0.1)	15,546.6 ± 10,208.0 [8472.9–22,620.2]	9582.5 ± 6160.2 [5313.7–13,851.2]
	Infected five times *, 3 (0.05)	26,536.3 ± 5967.2 [19,783.9–33,288.7]	13,210.8 ± 4631.6 [7969.8–18,451.9]
	Infected six times *, 2 (0.03)	33,541.9 ± 3671.5 [28,453.5–38,630.3]	16,114.5 ± 4991.5 [9196.8–23,032.2]
Disease severity (%)	Sporadic *, 288 (5.0)	14,440.3 ± 11,020.8 [13,167.5–15,713.15]	13,364.8 ± 11,630.2 [12,021.6–14,708.0]
	Episodic *, 3402 (58.4)	14,737.4 ± 10,530.3 [14,383.5–15,091.24]	12,937.9 ± 10,029.4 [12,600.9–13,274.9]
	Recurrent *, 1588 (27.2)	15,754.9 ± 15,982.5 [14,968.8–16,541]	12,700.4 ± 12,475.5 [12,086.9–13,314.0]
	Frequent *, 484 (8.3)	16,318.0 ± 9901.2 [15,435.9–17,200.07]	11,829.4 ± 7853.0 [11,129.7–12,529.0]
	Persistent *, 67 (1.1)	25,622.6 ± 32,305.3 [17,887.2–33,358.08]	11,746.6 ± 10,014.2 [9348.7–14,144.5]
Illness treatment	No treatment *, 1044 (17.9)	16,285.8 ± 17,820.8 [15,204.8–17,366.8]	14,120.2 ± 15,578.0 [13,175.2–15,065.1]
	Home remedies *, 3614 (62.0)	14,801.6 ± 10,717.0 [14,452.2–15,151.0]	12,555.4 ± 9281.5 [12,252.8–12,858.0]
	Prescribed medication *, 1085 (18.6)	15,755.6 ± 13,082.3 [14,977.2–16,534.1]	12,760.4 ± 9244.3 [12,210.3–13,310.4]
	Hospital admission *, 62 (1.1)	14,937.3 ± 9040.7 [12,687.0–17,187.7]	7207.1 ± 6178.3 [5669.2–8745.0]
	ICU admission *, 24 (0.4)	17,184.5 ± 13,057.7 [11,960.4–22,408.6]	5668.8 ± 4716.8 [3781.7–7555.9]

* *p* ≤ 0.05, significant difference between the PRE and POST conditions. ^†^ The disease severity was calculated using the prevalence and frequency of occurrence of predefined SARS-CoV-2 infection symptoms (1 = sporadic (1–20% of the time) to 5 = persistent (81–100% of the time)). Abbreviations: CI, confidence interval; ICU, intensive care unit; MET, metabolic equivalent task (1 MET = 3.5 mL O_2_·kg^−1^·min^−1^); mRNA, messenger ribonucleic acid; PA, physical activity; POST, one month post-SARS-CoV-2 infection; PRE, 1–2 wk pre-SARS-CoV-2 infection; PS, protein subunit; SARS-CoV-2, severe acute respiratory syndrome coronavirus 2; SD, standard deviation; VV, viral vector.

**Table 2:** Physical activity statistics by the daily occupation, transportation (to and from daily occupation), leisure-time, and regular sporting activity domains, presented as the means ± SD [95% CI] according to respondent vaccination profile, vaccine type(s) received, and incidence of SARS-CoV-2 re-infections under the PRE and POST conditions.

Variable	Domain	Subgroup, Frequency (%)	PRE, (MET-Min·Week^−1^)	POST, (MET-Min·Week^−1^)
	Daily occupation activities	All respondents *, 5829 (100.0)	4817.1 ± 3825.8 [4718.9–4915.3]	4245.6 ± 3587.1 [4153.5–4337.7]
	Transportation activities	All respondents *, 5829 (100.0)	1160.0 ± 1102.6 [1131.7–1188.3]	1003.3 ± 1051.6 [976.3–1030.3]
	Leisure-time activities	All respondents *, 5829 (100.0)	7307.5 ± 7439.1 [7116.5–7498.4]	6112.3 ± 6714.3 [5939.9–6284.6]
	Sporting activities	All respondents *, 5829 (100.0)	1971.7 ± 6498.0 [1804.9–2138.5]	1427.4 ± 4588.3 [1309.6–1545.2]
Pre-illness vaccination profile	Daily occupation activities	Unvaccinated *, 1222 (21.0)	4980.3 ± 3959.6 [4758.2–5202.3]	4581.0 ± 3785.8 [4368.7–4793.2]
		Partially vaccinated *, 2421 (41.5)	4815.3 ± 3791.3 [4664.3–4966.4]	4161.3 ± 3463.9 [4065.7–4349.0]
		Fully vaccinated *, 2186 (37.5)	4727.8 ± 3786.3 [4569.1–4886.5]	4151.5 ± 3597.7 [3952.0–4244.8]
		Vaccinated *, 4607 (79.0)	4773.8 ± 3788.8 [4664.4–4883.2]	4156.6 ± 3527.6 [4054.8–4258.5]
	Transportation activities	Unvaccinated *, 1222 (21.0)	1223.3 ± 1275.3 [1151.8–1294.8]	1115.4 ± 1258.5 [1044.9–1186.0]
		Partially vaccinated *, 2421 (41.5)	1137.0 ± 1051.1 [1095.2–1178.9]	947.8 ± 937.4 [944.7–1023.6]
		Fully vaccinated *, 2186 (37.5)	1150.0 ± 1052.3 [1105.9–1194.15]	1002.2 ± 1039.6 [920.3–1002.7]
		Vaccinated *, 4607 (79.0)	1143.2 ± 1051.6 [1112.8–1173.5]	973.6 ± 987.5 [945.1–1002.1]
	Leisure-time activities	Unvaccinated *, 1222 (21.0)	7941.3 ± 9004.73 [7436.4–8446.2]	6742.3 ± 7993.2 [6294.2–7190.5]
		Partially vaccinated *, 2421 (41.5)	7000.5 ± 6561.76 [6739.1–7261.9]	5726.3 ± 5824.6 [5590.5–6067.3]
		Fully vaccinated *, 2186 (37.5)	7293.2 ± 7367.38 [6984.3–7602.0]	6187.5 ± 6824.4 [5797.7–6359.3]
		Vaccinated *, 4607 (79.0)	7139.4 ± 6956.44 [6938.5–7340.2]	5945.1 ± 6322.3 [5762.6–6127.7]
	Sporting activities	Unvaccinated *, 1222 (21.0)	2510.3 ± 9320.65 [1987.7–3032.9]	1936.9 ± 7356.9 [1524.4–2349.3]
		Partially vaccinated *, 2421 (41.5)	1858.6 ± 6739.28 [1590.2–2127.1]	1149.5 ± 3301.3 [1094.3–1370.4]
		Fully vaccinated *, 2186 (37.5)	1795.8 ± 3683.21 [1641.4–1950.2]	1450.3 ± 3688.6 [1213.2–1508.7]
		Vaccinated *, 4607 (79.0)	1828.8 ± 5504.46 [1669.9–1987.8]	1292.2 ± 3493.3 [1191.4–1393.1]
Pre-illness vaccine type(s) received	Daily occupation activities	No vaccine *, 1222 (21.0)	4980.3 ± 3959.6 [4758.2–5202.3]	4580.9 ± 3785.8 [4368.7–4793.2]
		mRNA *, 3732 (64.0)	4733.1 ± 3750.1 [4612.8–4853.5]	4126.9 ± 3484.5 [4015.1–4238.7]
		VV *, 226 (3.9)	5643.3 ± 4474.0 [5060.0–6226.6]	4836.1 ± 3974.4 [4317.9–5354.3]
		PS, 4 (0.1)	4487.2 ± 2811.8 [1731.7–7242.8]	3917.2 ± 2639.1 [1331.0–6503.5]
		Combination of vaccines *, 645 (11.1)	4706.2 ± 3725.9 [4418.7–4993.7]	4092.0 ± 3596.8 [3814.4–4369.6]
	Transportation activities	No vaccine *, 1222 (21.0)	1223.3 ± 1275.3 [1151.8–1294.8]	1115.4 ± 1258.5 [1044.9–1186.0]
		mRNA *, 3732 (64.0)	1141.7 ± 1067.3 [1107.4–1175.9]	961.1 ± 978.0 [929.7–992.4]
		VV *, 226 (3.9)	1138.6 ± 913.1 [1019.6–1257.6]	1035.1 ± 933.2 [913.5–1156.8]
		PS *, 4 (0.1)	763.4 ± 234.7 [533.4–993.4]	250.5 ± 222.4 [32.5–468.5]
		Combination of vaccines *, 645 (11.1)	1156.1 ± 1008.2 [1078.3–1233.9]	1029.1 ± 1058.0 [947.4–1110.7]
	Leisure-time activities	No vaccine *, 1222 (21.0)	7941.3 ± 9004.7 [7436.4–8446.2]	6742.3 ± 7993.2 [6294.2–7190.5]
		mRNA *, 3732 (64.0)	7243.6 ± 6988.0 [7019.4–7467.8]	6049.3 ± 6467.3 [5841.8–6256.8]
		VV *, 226 (3.9)	6183.3 ± 6054.5 [5393.9–6972.6]	5026.6 ± 4655.0 [4419.7–5633.5]
		PS, 4 (0.1)	10,284.3 ± 3639.5 [6717.6–13,851.0]	8427.7 ± 2490.7 [5986.9–10,868.6]
		Combination of vaccines *, 645 (11.1)	6851.5 ± 7056.9 [6306.9–7396.1]	5648.8 ± 5956.2 [5189.1–6108.5]
	Sporting activities	No vaccine *, 1222 (21.0)	2510.3 ± 9320.6 [1987.7–3032.9]	1936.9 ± 7356.9 [1524.4–2349.3]
		mRNA *, 3732 (64.0)	1870.7 ± 5963.5 [1679.3–2062.0]	1287.3 ± 3600.2 [1171.7–1402.8]
		VV *, 226 (3.9)	1584.1 ± 2452.7 [1264.3–1903.9]	1281.1 ± 1992.7 [1021.3–1540.9]
		PS, 4 (0.1)	2678.3 ± 1516.5 [1192.2–4164.5]	810.5 ± 538.6 [282.7–1338.4]
		Combination of vaccines *, 645 (11.1)	1667.3 ± 2915.0 [1442.4–1892.3]	1327.9 ± 3285.3 [1074.4–1581.5]
Incidence of SARS-CoV-2 re-infections (frequency)	Daily occupation activities	Infected one time *, 4093 (70.2)	4682.5 ± 3812.8 [4565.7–4799.3]	4159.4 ± 3568.0 [4050.1–4268.7]
		Infected two times *, 1552 (26.6)	5115.5 ± 3811.2 [4925.9–5305.1]	4394.7 ± 3582.7 [4216.4–4572.9]
		Infected three times, 171 (2.9)	5303.3 ± 4096.0 [4689.4–5917.2]	5058.8 ± 4001.8 [4459.0–5658.6]
		Infected four times, 8 (0.1)	5357.6 ± 3924.0 [2638.5–8076.8]	2872.9 ± 2484.0 [1151.6–4594.2]
		Infected five times, 3 (0.05)	5775.0 ± 5649.3 [0.0–12,167.7]	3150.0 ± 2875.8 [0.0–6404.2]
		Infected six times, 2 (0.03)	3532.5 ± 922.8 [2253.6–4811.4]	2542.5 ± 2322.8 [0.0–5761.7]
	Transportation activities	Infected one time *, 4093 (70. 2)	1144.5 ± 1096.1 [1110.9–1178.1]	984.2 ± 1037.7 [952.4–1015.9]
		Infected two times *, 1552 (26.6)	1159.1 ± 1085.8 [1105.1–1213.1]	1017.6 ± 1076.5 [964.1–1071.2]
		Infected three times *, 171 (2.9)	1478.7 ± 1317.3 [1281.2–1676.1]	1291.4 ± 1113.5 [1124.5–1458.3]
		Infected four times, 8 (0.1)	1468.4 ± 1251.5 [601.1–2335.6]	964.5 ± 218.7 [813.0–1116.0]
		Infected five times, 3 (0.05)	2486.7 ± 1059.0 [1288.4–3685.1]	2116.3 ± 831.3 [1175.6–3057.0]
		Infected six times *, 2 (0.03)	3060.0 ± 594.0 [2236.8–3883.2]	3030.0 ± 551.5 [2265.6–3794.4]
	Leisure-time activities	Infected one time *, 4093 (70.2)	7283.2 ± 7450.3 [7055.0–7511.5]	6180.8 ± 6872.0 [5970.3–6391.4]
		Infected two times *, 1552 (26.6)	7321.0 ± 7351.3 [6955.3–7686.8]	6008.5 ± 6388.1 [5690.7–6326.3]
		Infected three times *, 171 (2.9)	7877.5 ± 8091.6 [6664.7–9090.3]	5642.9 ± 5915.5 [4756.3–6529.5]
		Infected four times, 8 (0.1)	5903.6 ± 5863.6 [1840.4–9966.8]	3733.0 ± 2491.3 [2006.7–5459.4]
		Infected five times, 3 (0.05)	8215.5 ± 5441.3 [2058.2–14,372.9]	2301.6 ± 1537.9 [561.4–4041.8]
		Infected six times *, 2 (0.03)	1934.7 ± 362.3 [1432.6–2436.8]	1647.0 ± 356.4 [1153.1–2140.9]
	Sporting activities	Infected one time *, 4093 (70.2)	1934.3 ± 7242.5 [1712.4–2156.2]	1395.0 ± 4675.2 [1251.8–1538.3]
		Infected two times *, 1552 (26.6)	1927.3 ± 4056.4 [1725.5–2129.1]	1388.4 ± 3701.8 [1204.2–1572.6]
		Infected three times, 171 (2.9)	2818.6 ± 5098.9 [2054.4–3582.9]	2366.6 ± 8258.9 [1128.7–3604.4]
		Infected four times, 8 (0.1)	2817.0 ± 2509.9 [1077.7–4556.2]	2012.1 ± 2016.7 [614.6–3409.6]
		Infected five times *, 3 (0.05)	10,059.0 ± 5194.2 [4181.3–15,936.8]	5642.9 ± 4757.8 [259.1–11,026.8]
		Infected six times *, 2 (0.03)	25,014.7 ± 1792.5 [22,530.5–27,499.0]	8895.0 ± 1760.7 [6454.8–11,335.2]

* *p* ≤ 0.05, significant difference between the PRE and POST conditions. Abbreviations: CI, confidence interval; MET, metabolic equivalent task (1 MET = 3.5 mL O_2_·kg^−1^·min^−1^); mRNA, messenger ribonucleic acid; POST, one month post-SARS-CoV-2 infection; PRE, 1–2 wk pre-SARS-CoV-2 infection; PS, protein subunit; SARS-CoV-2, severe acute respiratory syndrome coronavirus 2; SD, standard deviation; VV, viral vector.

**Table 3:** Overall PA estimates ^§^ presented as means ± SE [95% CI] under the POST condition according to respondent sex at birth, age, body mass index, PA level, smoking status, region of residence, education level, number of underlying medical conditions, vaccination profile, vaccine type(s) received, incidence of SARS-CoV-2 re-infections, disease severity ^†^, and illness treatment, adjusted for the covariate ^‡^ values of the PRE condition.

Variable	No.	Subgroup, Frequency (%)	PA (MET-Min·Week^−1^)	Significance
Sex at birth	1	Males, 1962 (33.7)	12,879.0 ± 167.6 [12,550.4–13,207.5]	NS
	2	Females, 3867 (66.374)	12,742.7 ± 119.4 [12,508.7–12,976.7]	
Age (yr)	1	Young (18–29), 464 (8.0)	11,832.3 ± 344.1 [11,157.6–12,506.9]	* 1 < 2,3
	2	Adults (30–49), 3074 (52.74)	12,868.7 ± 133.7 [12,606.6–13,130.8]	
	3	Middle-aged adults (50–59), 1959 (33.6)	13,061.9 ± 167.5 [12,733.6–13,390.3]	
	4	Old adults (60–69), 328 (5.6)	11,776.7 ± 409.3 [10,974.3–12,579.1]	* 4 < 3
	5	70+ (≥70), 4 (0.1)	----	
Body mass index (kg·m^−2^)	1	Underweight (<18.5), 112 (1.9)	12,179.4 ± 701.6 [10,803.9–13,554.8]	NS
	2	Acceptable weight (18.5–24.9), 2556 (43.8)	12,910.4 ± 146.8 [12,622.6–13,198.3]	
	3	Overweight (25.0–29.9), 2081 (35.7)	12,741.2 ± 162.7 [12,422.2–13,060.2]	
	4	Obese (≥30), 1080 (18.5)	12,654.6 ± 225.9 [12,211.7–13,097.5]	
PA level (MET-min·week^−1^)	1	Inactive (0), 1909 (32.7)	12,791.9 ± 171.8 [12,455.1–13,128.7]	
	2	Low PA (0–499), 1073 (18.4)	12,456.6 ± 227.8 [12,010.0–12,903.2]	
	3	Moderate PA (500–1000), 710 (12.2)	12,212.4 ± 278.9 [11,665.6–12,759.2]	* 3 < 4
	4	High PA (>1000), 2137 (36.7)	13,143.7 ± 165.9 [12,818.3–13,469.0]	
Smoking status	1	Never smoker, 3010 (51.6)	12,796.1 ± 135.3 [12,530.8–13,061.3]	NS
	2	Former smoker, 1056 (18.1)	12,836.1 ± 228.5 [12,388.1–13,284.0]	
	3	Someday smoker, 583 (10.0)	12,831.8 ± 307.4 [12,229.1–13,434.5]	
	4	Every day smoker, 1180 (20.2)	12,705.5 ± 216.1 [12,281.9–13,129.1]	
Region of residence	1	Urban region, 4184 (71.8)	12,909.6 ± 114.7 [12,684.7–13,134.5]	NS
	2	Peri-urban region, 1358 (23.3)	12,427.0 ± 201.4 [12,032.2–12,821.9]	
	3	Rural or off-the-grid region, 287 (4.9)	12,734.2 ± 438.0 [11,875.7–13,592.8]	
Education level (certificate)	1	Primary school certificate or lower, 5 (0.1)	----	NS
	2	Lower secondary school certificate, 60 (1.0)	14,845.2 ± 958.0 [12,967.2–16,723.3]	
	3	Upper secondary school certificate, 660 (11.3)	13,226.6 ± 289.1 [12,659.9–13,793.3]	
	4	Post-secondary school certificate, 428 (7.3)	12,617.4 ± 358.6 [11,914.3–13,320.5]	
	5	Bachelor degree, 2271 (39.0)	12,768.8 ± 155.7 [12,463.4–13,074.1]	
	6	MSc/master’s degree, 2120 (36.4)	12,712.6 ± 161.2 [12,396.6–13,028.6]	
	7	PhD/doctorate, 285 (4.9)	12,326.4 ± 439.9 [11,464.0–13,188.8]	
Number of underlying medical conditions	1	No conditions, 2410 (41.3)	13,000.6 ± 151.0 [12,704.6–13,296.6]	
	2	1 condition, 2858 (49.0)	12,847.9 ± 138.6 [12,576.2–13,119.7]	
	3	2–5 conditions, 539 (9.2)	11,631.6 ± 319.3 [11,005.7–12,257.5]	* 3 < 1,2
	4	6–10 conditions, 22 (0.4)	10,190.2 ± 1580.3 [7092.2–13,288.1]	
	5	≥10 conditions, 0 (0.00)	----	
Pre-illness vaccination profile	1	Unvaccinated, 1222 (21.0)	13,533.0 ± 212.1 [13,117.2–13,948.9]	
	2	Partially vaccinated, 2421 (41.5)	12,252.9 ± 150.5 [11,957.8–12,548.0]	* 2 < 1,3
	3	Fully vaccinated, 2186 (37.5)	12,965.7 ± 158.4 [12,655.1–13,276.2]	
	4	Vaccinated, 4607 (79.0)	12,591.2 ± 109.2 [12,377.0–12,805.3]	* 4 < 1
Pre-illness vaccine type(s) received	1	No vaccine, 1222 (21.0)	13,532.8 ± 212.3 [13,116.5–13,949.1]	
	2	mRNA, 3732 (64.0)	12,585.5 ± 121.4 [12,347.6–12,823.5]	* 2 < 1
	3	VV, 226 (3.9)	12,605.0 ± 493.2 [11,638.2–13,571.8]	
	4	PS, 4 (0.1)	----	
	5	Combination of vaccines, 645 (11.1)	12,625.0 ± 292.0 [12,052.6–13,197.4]	
Incidence of SARS-CoV-2 re-infections (frequency)	1	Infected one time, 4093 (70.2)	12,847.5 ± 116.0 [12,620.1–13,074.8]	NS
	2	Infected two times, 1552 (26.6)	12,647.9 ± 188.3 [12,278.7–13,017.1]	
	3	Infected three times, 171 (2.9)	13,016.2 ± 567.7 [11,903.3–14,129.0]	
	4	Infected four times, 8 (0.1)	----	
	5	Infected five times, 3 (0.05)	----	
	6	Infected six times, 2 (0.03)	----	
Disease severity (%)	1	Sporadic, 288 (4.9)	13,864.1 ± 433.3 [13,014.8–14,713.5]	
	2	Episodic, 3402 (58.4)	13,255.4 ± 126.1 [13,008.2–13,502.6]	
	3	Recurrent, 1588 (27.2)	12,395.3 ± 184.5 [12,033.6–12,757.1]	* 3 < 1,2
	4	Frequent, 484 (8.3)	11,179.7 ± 334.3 [10,524.4–11,835.0]	* 4 < 1,2,3
	5	Persistent, 67 (1.1)	5403.2 ± 901.6 [3635.7–7170.7]	* 5 < 1,2,3,4
Illness treatment	1	No treatment, 1044 (17.9)	13,498.5 ± 228.4 [13,050.7–13,946.3]	
	2	Home remedies, 3614 (62.0)	12,830.0 ± 122.7 [12,589.3–13,070.6]	
	3	Prescribed medication, 1085 (18.6)	12,458.8 ± 224.0 [12,019.8–12,897.9]	* 3 < 1
	4	Hospital admission, 62 (1.1)	7399.7 ± 936.8 [5563.3–9236.1]	* 4 < 1,2,3
	5	ICU admission, 24 (0.4)	4504.4 ± 1505.7 [1552.7–7456.1]	* 5 < 1,2,3

^§^ Estimates of the “70+”, “primary school certificate or lower”, “≥10 conditions”, “PS-type vaccine”, “infected four times”, “infected five times”, and “infected six times” subgroups (due to their small sample sizes) were not practically significant, and therefore were not included in the analysis. ^†^ The disease severity was calculated using the prevalence and frequency of occurrence of predefined SARS-CoV-2 infection symptoms (1 = sporadic (1–20% of the time) to 5 = persistent (81–100% of the time)). ^‡^ The value of 15,256.3 MET-min·week^−1^ (PRE value) was used to evaluate covariates in the model. * *p* ≤ 0.05, significant effect of SARS-CoV-2 infection on PA subgroups. Abbreviations: CI, confidence interval; ICU, intensive care unit; MET, metabolic equivalent task (1 MET = 3.5 mL O_2_ kg^−1^·min^−1^); mRNA, messenger ribonucleic acid; NS, no significance; PA, physical activity; POST, one month post-SARS-CoV-2 infection; PRE, 1–2 wk pre-SARS-CoV-2 infection; PS, protein subunit; SARS-CoV-2, severe acute respiratory syndrome coronavirus 2; SE, standard error; VV, viral vector.

**Table 4:** Daily occupation, transportation (to and from daily occupation), leisure-time, and regular sporting activity estimates ^§^ according to respondent vaccination profile, vaccine type(s) received, and incidence of SARS-CoV-2 re-infections, presented as means ± SE [95% CI] under the POST conditions and adjusted for the covariate values of the PRE condition.

Variable	Domain	No.	Subgroup, Frequency (%)	PA (MET-Min·Week^−1^)	Significance
Pre-illness vaccination profile	^†^ Daily occupation activities	1	Unvaccinated, 1222 (21.0)	4469.3 ± 70.0 [4332.0–4606.6]	
		2	Partially vaccinated, 2421 (41.5)	4162.5 ± 49.8 [4065.0–4260.0]	* 2 < 1
		3	Fully vaccinated, 2186 (37.5)	4212.5 ± 52.4 [4109.9–4315.2]	* 3 < 1
		4	Vaccinated, 4607 (79.0)	4186.2 ± 36.1 [4115.5–4256.9]	* 4 < 1
	^‡^ Transportation activities	1	Unvaccinated, 1222 (21.0)	1076.3 ± 22.9 [1031.5–1121.2]	
		2	Partially vaccinated, 2421 (41.5)	962.0 ± 16.3 [930.1–993.8]	* 2 < 1
		3	Fully vaccinated, 2186 (37.5)	1008.3 ± 17.1 [974.8–1041.9]	* 3 < 1
		4	Vaccinated, 4607 (79.0)	984.0 ± 11.8 [960.9–1007.1]	* 4 < 1
	^#^ Leisure-time activities	1	Unvaccinated, 1222 (21.0)	6378.2 ± 148.0 [6088.1–6668.3]	
		2	Partially vaccinated, 2421 (41.5)	5902.7 ± 105.1 [5696.7–6108.7]	* 2 < 1
		3	Fully vaccinated, 2186 (37.5)	6195.7 ± 110.6 [5978.9–6412.4]	
		4	Vaccinated, 4607 (79.0)	6041.8 ± 76.2 [5892.4–6191.1]	* 4 < 1
	^¶^ Sporting activities	1	Unvaccinated, 1222 (21.0)	1684.9 ± 98.1 [1492.5–1877.2]	
		2	Partially vaccinated, 2421 (41.5)	1202.4 ± 69.7 [1065.9–1339.0]	* 2 < 1,3
		3	Fully vaccinated, 2186 (37.5)	1532.5 ± 73.3 [1388.8–1676.3]	
		4	Vaccinated, 4607 (79.0)	1359.1 ± 50.5 [1260.0–1458.1]	* 4 < 1
Pre-illness vaccine type(s) received	^†^ Daily occupation activities	1	No vaccine, 1222 (21.0)	4469.4 ± 70.1 [4332.0–4606.7]	
		2	mRNA, 3732 (64.0)	4184.3 ± 40.1 [4105.7–4262.9]	* 2 < 1
		3	VV, 226 (3.9)	4271.1 ± 163.0 [3951.4–4590.6]	
		4	PS, 4 (0.1)	4142.8 ± 1224.4 [1742.6–6543.0]	
		5	Combination of vaccines, 645 (11.1)	4167.8 ± 96.4 [3978.8–4356.8]	
	^‡^ Transportation activities	1	No vaccine, 1222 (21.0)	1076.4 ± 22.9 [1031.5–1121.2]	
		2	mRNA, 3732 (64.0)	972.4 ± 13.1 [946.7–998.0]	* 2 < 1
		3	VV, 226 (3.9)	1048.3 ± 53.2 [944.0–1152.7]	
		4	PS, 4 (0.1)	----	
		5	Combination of vaccines, 645 (11.1)	1031.5 ± 31.5 [969.7–1093.2]	
	^#^ Leisure-time activities	1	No vaccine, 1222 (21.0)	6378.3 ± 148.0 [6088.1–6668.5]	NS
		2	mRNA, 3732 (64.0)	6086.0 ± 84.6 [5920.0–6251.9]	
		3	VV, 226 (3.9)	5672.4 ± 344.1 [4997.8–6347.0]	
		4	PS, 4 (0.1)	6717.9 ± 2585.7 [1648.9–11,786.8]	
		5	Combination of vaccines, 645 (11.1)	5910.7 ± 203.7 [5511.5–6309.9]	
	^¶^ Sporting activities	1	No vaccine, 1222 (21.0)	1684.9 ± 98.2 [1492.3–1877.4]	
		2	mRNA, 3732 (64.0)	1334.5 ± 56.2 [1224.4–1444.6]	* 2 < 1
		3	VV, 226 (3.9)	1462.5 ± 228.2 [1015.1–1909.9]	
		4	PS, 4 (0.1)	----	
		5	Combination of vaccines, 645 (11.1)	1470.3 ± 135.1 [1205.5–1735.2]	
Incidence of SARS-CoV-2 re-infections (frequency)	^†^ Daily occupation activities	1	Infected one time, 4093 (70.2)	4251.6 ± 38.3 [4176.5–4326.6]	NS
		2	Infected two times, 1552 (26.6)	4190.4 ± 62.2 [4068.4–4312.3]	
		3	Infected three times, 171 (2.9)	4725.9 ± 187.3 [4358.7–5093.1]	
		4	Infected four times, 8 (0.1)	----	
		5	Infected five times, 3 (0.05)	----	
		6	Infected six times, 2 (0.03)	----	
	^‡^ Transportation activities	1	Infected one time, 4093 (70.2)	993.7 ± 12.5 [969.2–1018.2]	NS
		2	Infected two times, 1552 (26.6)	1018.2 ± 20.3 [978.3–1058.0]	
		3	Infected three times, 171 (2.9)	1094.9 ± 61.3 [974.7–1215.1]	
		4	Infected four times, 8 (0.1)	----	
		5	Infected five times, 3 (0.05)	----	
		6	Infected six times, 2 (0.03)	----	
	^#^ Leisure-time activities	1	Infected one time, 4093 (70.2)	6194.8 ± 80.8 [6036.4–6353.2]	NS
		2	Infected two times, 1552 (26.6)	6000.7 ± 131.2 [5743.4–6258.0]	
		3	Infected three times, 171 (2.9)	5314.7 ± 395.4 [4539.5–6089.8]	
		4	Infected four times, 8 (0.1)	----	
		5	Infected five times, 3 (0.05)	----	
		6	Infected six times, 2 (0.03)	----	
	^¶^ Sporting activities	1	Infected one time, 4093 (70.2)	1412.6 ± 53.6 [1307.4–1517.7]	NS
		2	Infected two times, 1552 (26.6)	1409.2 ± 87.1 [1238.4–1580.0]	
		3	Infected three times, 171 (2.9)	1969.5 ± 262.5 [1454.8–2484.1]	
		4	Infected four times, 8 (0.1)	----	
		5	Infected five times, 3 (0.05)	----	
		6	Infected six times, 2 (0.03)	----	

^§^ Estimates for the “PS-type vaccine”, “infected four times”, “infected five times”, and “infected six times” subgroups (due to their small sample sizes) were not practically significant, and therefore were not included in the analysis. ^†^ The value of 4817.1 MET-min·week^−1^ (PRE value) was used to evaluate covariates in the model. ^‡^ The value of 1160.0 MET-min·week^−1^ (PRE value) was used to evaluate covariates in the model. ^#^ The value of 7307.5 MET-min·week^−1^ (PRE value) was used to evaluate covariates in the model. ^¶^ The value of 1971.7 MET-min·week^−1^ (PRE value) was used to evaluate covariates in the model. * *p* ≤ 0.05, significant effect of SARS-CoV-2 infection on PA subgroups. Abbreviations: CI, confidence interval; MET, metabolic equivalent task (1 MET = 3.5 mL O_2_·kg^−1^·min^−1^); mRNA, messenger ribonucleic acid; NS, no significance; PA, physical activity; POST, one month post-SARS-CoV-2 infection; PRE, 1–2 wk pre-SARS-CoV-2 infection; PS, protein subunit; SARS-CoV-2, severe acute respiratory syndrome coronavirus 2; SE, standard error; VV, viral vector.

## Data Availability

The raw data supporting the conclusions of this article will be made available by the corresponded author upon reasonable request once all relevant substudies have been reported and completed. The study protocol, data dictionary, and statistical analysis plan can also be made available by the corresponding author upon request.
